# Pharmacological modulation of fish-induced depth selection in *D. magna*: the role of cholinergic and GABAergic signalling

**DOI:** 10.1038/s41598-021-98886-w

**Published:** 2021-09-30

**Authors:** Juliette Bedrossiantz, Inmaculada Fuertes, Demetrio Raldua, Carlos Barata

**Affiliations:** Department of Environmental Chemistry, Institute of Environmental Assessment and Water Research, CSIC, Jordi Girobna 18, 08034 Barcelona, Spain

**Keywords:** Behavioural ecology, Neurophysiology

## Abstract

Animal behaviour is closely related to individual fitness, which allows animals to choose suitable mates or avoid predation. The central nervous system regulates many aspects of animal behaviour responses. Therefore, behavioural responses can be especially sensitive to compounds with a neurodevelopmental or neurofunctional mode of action. Phototactic behavioural changes against fish in the freshwater crustacean *Daphnia magna* have been the subject of many ecological investigations. The aim of this study was to identify which neurotransmitter systems modulate phototactic behaviour to fish kairomones. We used a positive phototactic *D. magna* clone (P_1_32,85) that shows marked negative phototactism after exposure to fish kairomones. Treatments included up to 16 known agonists and antagonists of the serotonergic, cholinergic, dopaminergic, histaminergic, glutamatergic and GABAergic systems. It was hypothesized that many neurological signalling pathways may modulate *D. magna* phototactic behaviour to fish kairomones. A new custom-designed device with vertically oriented chambers was used, and changes in the preferred areas (bottom, middle, and upper areas) were analysed using groups of animals after 24 h of exposure to the selected substance(s). The results indicated that agonists of the muscarinic acetylcholine and GABA_A_ receptors and their equi-effective mixture ameliorated the negative phototactic response to fish kairomones, whereas antagonists and their mixtures increased the negative phototactism to fish kairomones. Interestingly, inhibition of the muscarinic acetylcholine receptor abolished positive phototaxis, thus inducing the phototactic response to fish kairomones. Analysis of the profile of neurotransmitters and their related metabolites showed that the *D. magna* behavioural responses induced by fish depend on changes in the levels of acetylcholine, dopamine and GABA.

## Introduction

Chemical signals mediate many inter- and intra-specific interactions among aquatic animals^1^. Many prey organisms are able to detect the presence of predators and precisely respond to them, changing their phenotype. Inducible defences, when under an increased risk of predation, are key adaptive strategies of prey organisms, since these defence mechanisms increase the chance of survival when predation risks are high but reduce costs when predators are not present. Understanding the sensory mechanisms that are in line with the neuronal signalling pathways will significantly contribute comprehensive insight into how anthropogenically released endocrine disruptors will affect trophic interactions^2^.

A known ecologically relevant predator-induced defence response against fish in many zooplankton species is diel vertical migration^3^. Changes in phototactic behaviour in response to chemical signals released from predatory fish have been widely studied in the crustacean prey *Daphnia magna*, which is a keystone species in freshwater food webs and an established model organism in ecotoxicology and evolutionary ecology^4^. *D. magna* populations that have adapted to high fish predation pressures may have marked negative phototactic behaviours^5^ and/or additional anti-predator life history changes^6,7^. Chemical cues released by fish, so-called kairomones, are able to induce anti-predatory behavioural responses shortly after exposure in a concentration- and light intensity-dependent manner^8–10^.

Despite these known *D. magna* phenotypic responses, the underlying neuronal mechanisms for inducible defences against fish have been poorly investigated^2,11^.

Weiss et al.^11^ reported that the inhibitory effects of GABA decreased fish-induced life history changes in the related species *Daphnia pulex*, while the application of cholinergic stimulants had no effect when used in combination with fish-related cues. Studies conducted with other neurotransmitters showed that antihistamine compounds increased positive phototaxis towards UV radiation^12^ and that dopamine decreased locomotor activity in *D. magna*^13,14^. Psychiatric drugs such as fluoxetine, which is known to enhance serotonin, and carbamazepine and diazepam, increase positive phototactic behaviour in *D. magna* individuals^15^. *D. magna* CRISPR/Cas tryptophan hydrolase gene mutants lacking serotonin responded to light to a greater extent than wild-type individuals^16^. Nevertheless, to date, the studied neuronal stimulants have been able to modulate but not induce anti-predatory fish phenotypes, indicating a pathway of interlinked steps.

The scarcity of *Daphnia* neurophysiological studies for inducible defences against fish predation contrasts with those conducted for invertebrate predation. Ecological theory, with the support of several experimental studies, predicts that fish and invertebrate predation may induce opposite life history changes to favour smaller and larger *Daphnia* individuals, respectively^17–19^. Nevertheless, the reported neurophysiological mechanisms regulating inducible morphological changes in *Daphnia* exposed to invertebrate predators may help elucidate the mechanisms for fish predation. Neurophysiological studies have shown that inducible invertebrate anti-predatory defensive responses in *Daphnia* are highly variable, prey- and species-dependent and comprise different signalling components, such as the involvement of cholinergic, glutamatergic, dopaminergic and GABAergic signalling^11,20–22^. GABA_A_ and cholinergic receptor antagonists/activators such as picrotoxin and physostigmine enhance Chaoborus-induced neck tooth growth in *D. pulex*, whereas the anti-cholinergic compound atropine suppresses these effects^11,21^. Miyakawa et al.^20^ combined transcriptomics with the use of receptor activators and inhibitors and found that antagonists of ionotropic glutamate receptors reverted the formation of neck teeth in *D. pulex* co-exposed to phantom midge larvae kairomones, but agonists of these receptor types did not have any effect. Furthermore, cholinergic and dopaminergic activators alone were able to induce morphological defences similar to those of invertebrate predators in several *Daphnia* species, although the results varied across species^22,23^. Thus, the primary cholinergic, serotonergic, dopaminergic, histaminergic, GABAergic and glutamatergic neurological systems are candidate pathways to regulate the induced phototactic responses of *D. magna* to fish kairomones.

The above-mentioned neurophysiological studies, however, did not directly address how neurotransmitters and their related metabolite profiles changed upon exposure to predation cues. Neurotransmitters bind to specific receptors on the plasma membrane of postsynaptic cells, causing a change in their permeability to ions that can promote or inhibit the generation of an action potential in these postsynaptic cells, such as motor neurons. This means that the study of neurotransmitter profiles across predator treatments should provide valuable information on the molecular mechanisms involved in such responses. Unfortunately, few studies have related neurotransmitters to anti-predator defence responses in *Daphnia*. Immuno-histochemical studies have shown that drugs that modulate phototactic behaviour in *D. magna* may affect the neurotransmitter levels of serotonergic cells located in brain neurons^15,24^. There is also evidence of histamine-like labelling in the nervous system of *D. pulex* and antihistamine compounds could increase positive phototaxis towards UV irradiation^12^. Nonetheless, it is still uncertain how these neuronal systems determine the development of phenotypically plastic anti-predator defences.

Recently, the detection and quantification of a broader range of neurochemicals in *Daphnia* using liquid chromatography coupled with tandem mass spectrometry have become possible^25^. This methodology has allowed the reporting of several neuroactive drugs that act in *Daphnia* similarly to their action in humans. Fluoxetine, a selective serotonin reuptake inhibitor (SSRI), and chloro-DL-phenylalanine (PCPA), a tryptophan enzyme inhibitor, increased and decreased the whole-body levels of serotonin and their related degradation products, respectively, in *Daphnia;* and *6*-hydroxydopamine, which promotes the destruction of dopaminergic and noradrenergic neurons, reduced the levels of adrenergic metabolites^25,26^. Previous studies have also reported that some drugs act on neurotransmitters in an non-specific and unexpected way: diazepam, a positive allosteric modulator of GABA type A receptors (GABA_A_), propranolol, a non-selective β-receptor antagonist, and antihistaminergic drugs alter the concentrations of adrenergic metabolites.

The objective of the present work was to study the neurotransmitter signalling pathways involved in the phototactic responses of *D. magna* to fish kairomones. To achieve that objective, we monitored changes in the vertical positions of animals across dark and light periods following co-exposure to fish kairomone-conditioned water (hereafter referred to fish kairomones, FKs) and select agonists and antagonists using a previously developed high-throughput video-tracking platform^27^. Behavioural studies were then complemented with the analysis of up to 16 neurochemicals from eight neurotransmitter systems in *D. magna* individuals exposed to FKs and select compounds using HPLC–MS/MS following previously developed methods^25,26^. Phototactic behaviour assays using different FK concentrations in water and light intensities were performed before co-exposure with agonists and antagonists to select an optimal response to the FKs. Our initial hypothesis was that any of the eight tested neurotransmitter systems could be involved in and/or modulate the phototactic responses of *D. magna* to fish kairomones.

## Results

### Method optimization

The proposed behavioural device in this study is able to monitor the phototactic trajectories of the adult females of the studied clone. Adults of the tested clone were located predominantly in the top of the arena during the dark period, moved to the bottom during the first several minutes after the sudden turn on of the apical illumination, and then returned to the top afterwards. Exposure to fish kairomone-conditioned water for 24 h dramatically increased the number of individuals swimming close to the bottom. Changes in the percentage of individuals swimming on the top of the arena during the light period rather than the distance moved were the most distinctive behavioural response to FKs. Further details are depicted in the Supplementary Results section and in Figs. S1, S2, and S3.

### Phototactic behaviour

Approximately half of the reported data on phototactic behaviour meet ANOVA assumptions. Two-way nested ANOVA analyses of these data indicated non-significant (*P* ≥ 0.05) effects from the nested factor (arena) and significant (*P* < 0.05) effects from fish kairomones (FKs) in the many experiments that were performed (the results are reported in Supplementary Tables S3 and S5). This indicates that the experimental vessels or arena in which the animals (in groups of five or six individuals) were exposed and monitored did not affect their behaviour. As expected, FKs decreased the positive phototactic behaviour.

### Phototactic effects of the studied pharmaceuticals and FK

**Figure 1:**
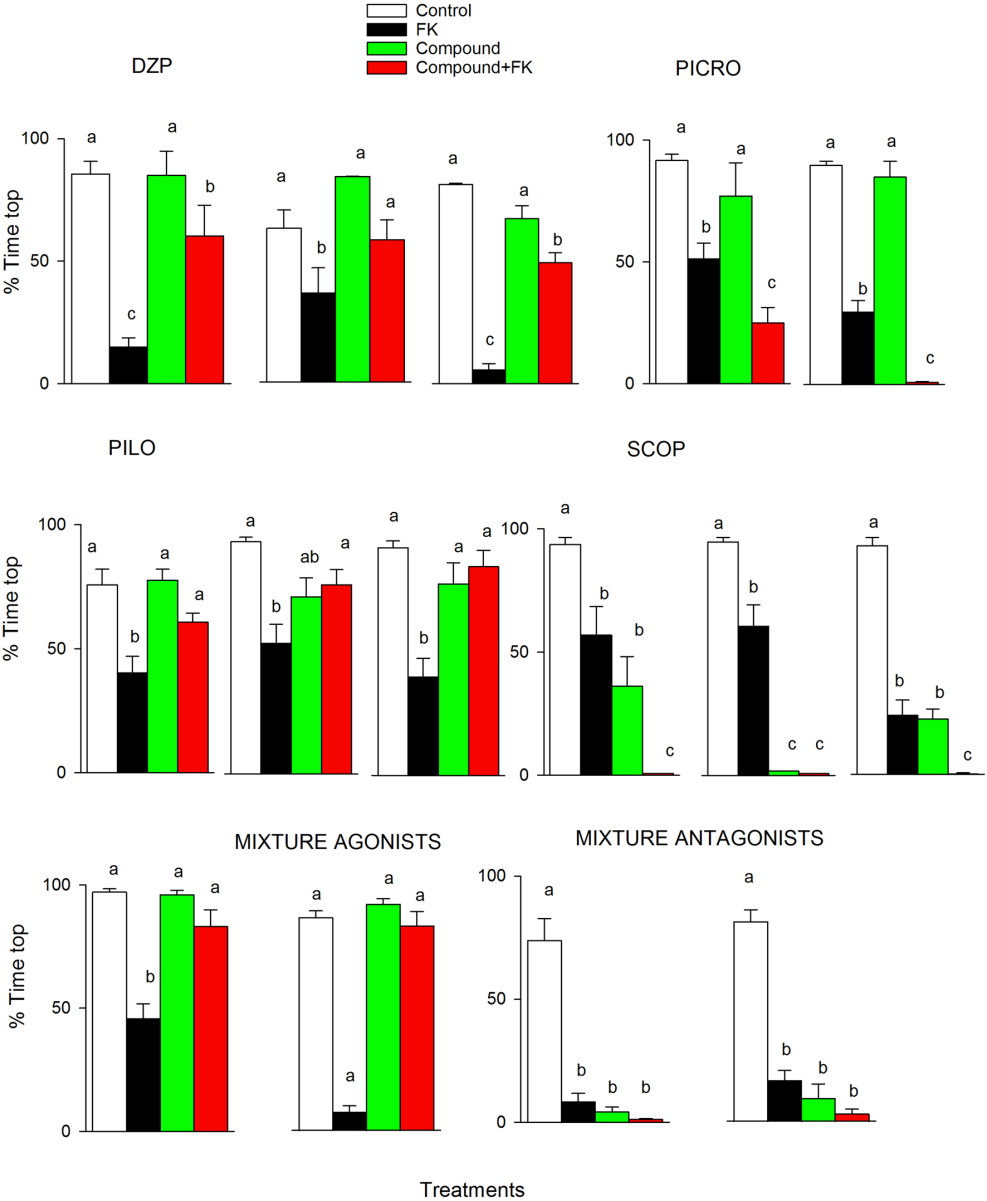
Agonists and antagonists of the muscarinic acetylcholine and GABA_A_ receptors and their mixtures consistently affected the response of adult females to FK. Percentage of individuals swimming in the top virtual zone during the late period of light (mean ± SE, N = 10–15) following exposure to FK, DZP, PICRO, PILO, SCOP or binary mixtures of agonists and antagonists. Grouped graphs depict the results from the two or three independent experiments performed. Complete phototactic trajectories are shown in Figs. S4 and S5 in the Supplementary Materials. Within each graph, different letters indicate significant (*P* < 0.05) differences following parametric or non-parametric one-way ANOVA and multiple comparisons tests.

Four out of the 16 studied compounds, including agonists and antagonists of the muscarinic acetylcholine (pilocarpine-PILO, scopolamine-SCOP) and GABA_A_ receptors (diazepam-DZP, picrotoxin-PICRO), consistently significantly (*P* < 0.05) affected the adult female response to FKs across the two or three experiments performed (Fig. 1). The non-parametric and parametric ANOVA results are depicted in Supplementary Tables S4 and S5, respectively. Agonists (PILO and DZP) ameliorated the effects of the FKs, whereas antagonists increased these effects. Furthermore, binary equi-effective combinations of GABAergic and cholinergic agonists (DZP and PILO dosed at 50 and 500 μg/L, respectively) and antagonists (PICRO and SCOP dosed at 0.5 and 50 μg/L, respectively) acted similarly to their individual constituents (Fig. 1). Mixtures of agonists ameliorated the effects of the FKs, whereas mixtures of antagonists enhanced the FK effects.

**Figure 2:**
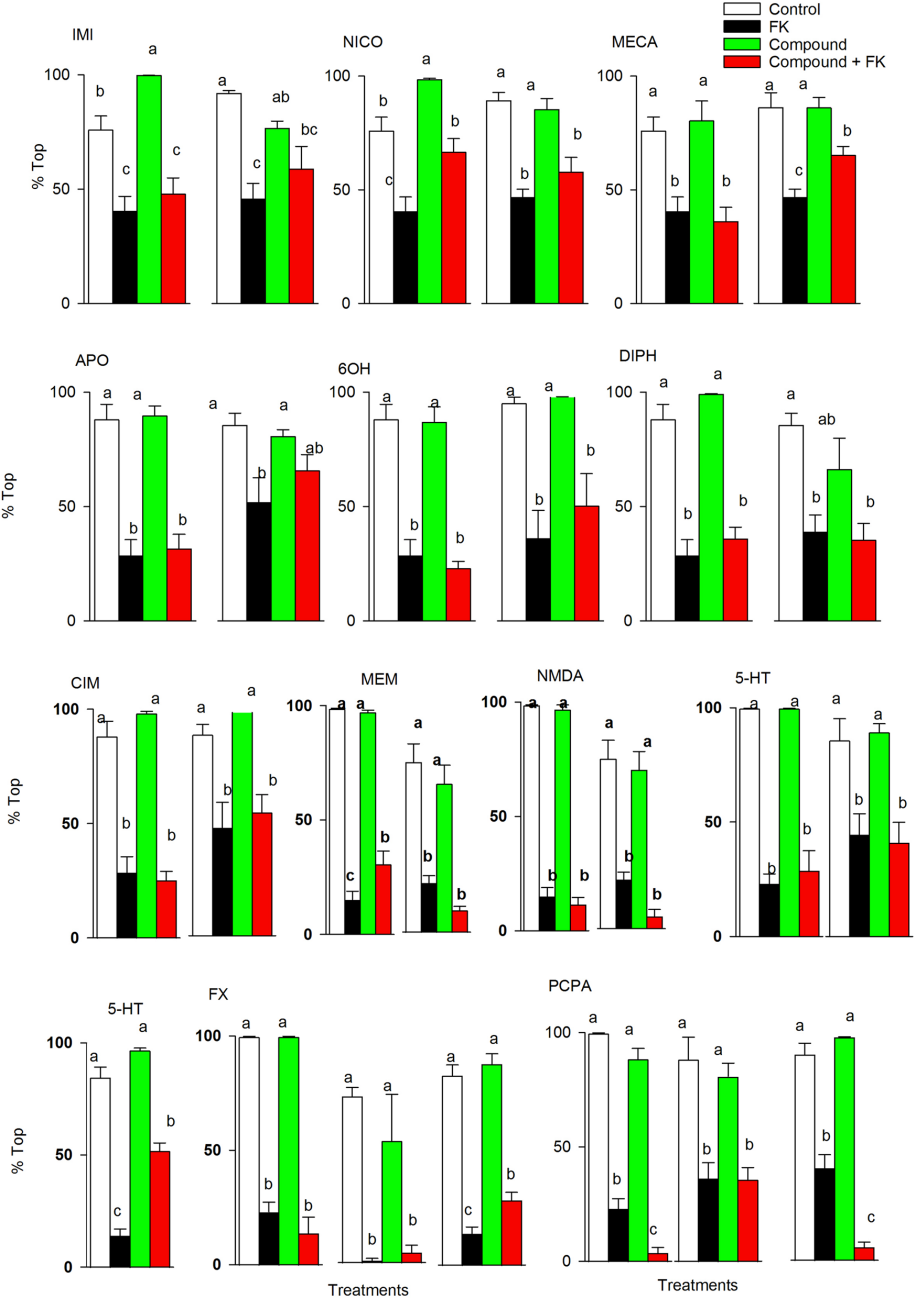
Inhibition and stimulation of the nicotinic acetyl cholinergic, serotonergic, dopaminergic, histaminergic and glutamatergic signalling pathways only marginally modulate phototactic responses to FK. Percentage of individuals swimming in the top virtual zone during the late period of light (mean ± SE, N = 10–15) across the remaining 12 serotonergic, histaminergic, dopaminergic and glutamatergic compounds. The results from the two or three experiments performed are depicted. Complete phototactic trajectories are shown in Supplementary Figs. S5 and S6. Within each graph, different letters indicate significant (*P* < 0.05) differences following parametric or non-parametric ANOVA and multiple comparisons tests.

Among the remaining test compounds, the effects of nicotinic agonists (nicotine-NICO, imidacloprid-IMI) and an antagonist (mecamylamine-MECA) were unclear (Fig. 2).

In only one of the three experiments performed, NICO, IMI and MECA ameliorated the FK effects irrespective of their opposite mode of action. The dopaminergic agonists apomorphine (APO) ameliorate the effects of FK on phototactic behaviour in only one out of the two experiments performed (Fig. 2). A serotonergic agonist (serotonin-5-HT) or a compound that increased serotonin levels (fluoxetine-FX) ameliorated the FK effects in one out of three experiments, whereas chloro-DL-phenylalanine (PCPA), which inhibits the activity of tryptophan hydrolase and hence decreases serotonin levels, increased the effects of FKs in two out of three experiments (Fig. 2). The rest of the tested compounds with dopaminergic and adrenergic (6-hydroxydopamine-6OH), histaminergic (diphenhydramine-DIPH, cimetidine-CIM) and glutamatergic (memantine-MEM, N-methyl-D-aspartic acid-NMDA) activities did not alter the FK effects on phototactic behaviour (Fig. 2). More information on the phototactic time responses of the 16 studied compounds and mixtures is shown in Supplementary Figs. S4, S5, S6 and S7.

### Metabolites

**Figure 3:**
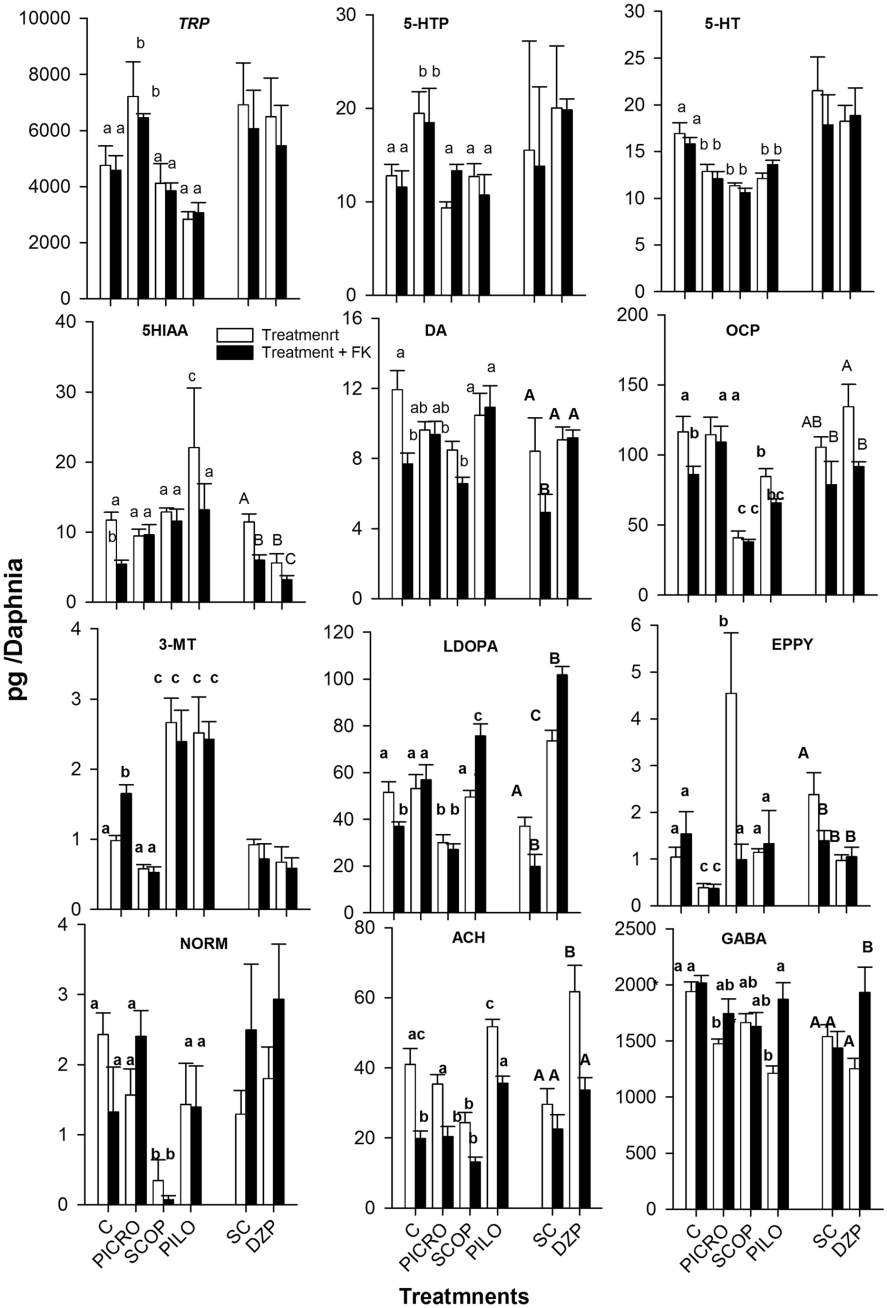
Up to 12 out of the 17 analysed neurotransmitters and related metabolites responded to the studied treatments. Selected neurotransmitters and their related metabolite concentrations (mean ± SE, N = 5–10) in the whole-body tissues of *D. magna* females exposed to the studied chemical treatments. The results are from two different experiments. Experiment 1 included the control (C), PICRO, SCOP, and PILO, and experiment 2 included a solvent control (SC) and DZP treatments; the results are depicted together. Within each graph, different letters indicate significant (*P* < 0.05) differences following ANOVA and multiple Tukey’s multiple comparisons tests. To avoid confusion, letters differentiating the DZP experiment are in upper case. The lack of any letter indicates non-significant (*P* ≥ 0.05) differences.

We quantified up to 17 metabolites belonging to seven different neurotransmitter systems^26^. The concentrations of 12 out of 17 metabolites varied significantly among the tested compounds within and across FK treatments (Fig. 3; ANOVA results are depicted in Supplementary Table S6). The metabolite profiles obtained from the two sets of experiments for the control, solvent controls and FK treatments were quite consistent for at least 9 metabolites (TRP, 5-HTP, 5-HT, 5-HIAA, DA, OCP, L-DOPA, ACH, GABA; Fig. 3). Exposure to FKs alone decreased the concentrations of 5-HIAA, DA, OCT, L-DOPA and ACH (Fig. 3). The tested antagonists SCOP and PICRO had similar effects, decreasing the concentrations of 5-HT (serotonin), DA and GABA. Alternatively, the studied agonists PILO and DZP enhanced ACH levels. GABAergic compounds decreased the concentration of EPPY, and cholinergic compounds decreased the level of OCP and increased the level of 3-MT. Compound-specific effects included increased levels of TRP and 5-HTP and a decreased concentration of 3-MT by PICRO; an increased concentration of EPPY and decreased levels of NORM and ACH by SCOP; and an enhanced level of 5-HIAA by PILO. Significant (*P* < 0.05) interaction terms between FKs and drug treatments were observed for 5-HIAA, DA, LDOPA, EPPY, GABA and ACH (Supplementary Table S6). The studied agonists PILO and DZP increased L-DOPA and GABA contents upon co-exposure to FKs. The effects of FKs on the decrease in ACH were ameliorated by co-exposure to the agonists PILO and DZP. The effects of the FKs on DA decreased after co-exposure to the antagonists PICRO and PILO. DZP alone and when co-exposed with FKs decreased the level of 5-HIAA. FKs reduced the level of EPPY relative to control treatments in females exposed to SCOP and DZP.

**Figure 4:**
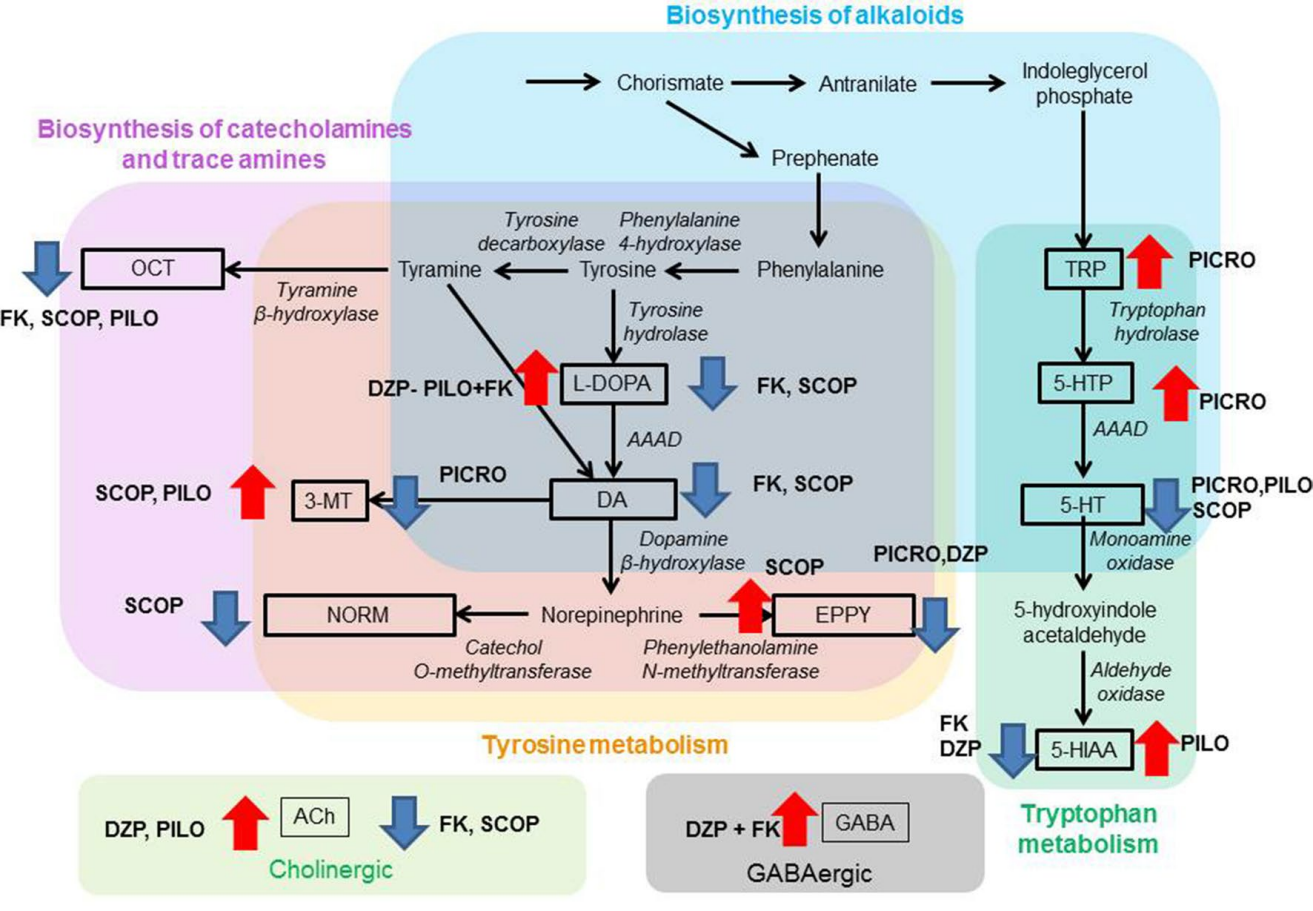
Affected metabolites depicted in KEGG pathways following Fuertes et al.^26^. Black-boxed metabolites are those that show significant treatments differences. In italics, the catalytic enzymes of the reactions of interest are detailed. Arrows and the compounds associated to them indicate the direction of the observed treatment effect according to Fig. 3.

The schematic representation of the affected metabolites within the KEGG neurotransmitter pathways is shown in Fig. 4, which may help to clarify drug effects in the metabolism of catecholamines and serotonergic metabolites. PICRO reduced 5-HT probably reducing its intermediary 5-HTP and its primary source TRP. PILO had the opposite metabolic effect, reducing 5-HT by increasing its metabolism towards 5-HIAA. FK and SCOP reduced DA and its intermediary L-DOPA. SCOP also increase the level of the DA metabolite 3-MT and of EPPY, which is synthetized from DA.

## Discussion

### Phototactic behaviour

The optimization results of the proposed behavioural setup allowed the phototactic behaviour of the studied *D. magna* clone and the effects of FK treatment on this behaviour to be monitored and quantified. Furthermore, the effects of the FKs were evident only upon light exposure, were more apparent after a short (5 min) acclimation to light and were tightly regulated by the light intensity. The above-mentioned factors agree with previous studies, which found a marked positive phototactism of clone P_1_32,85^28^ and that the effects of the FKs become consistent after 5 min of light exposure^28^. Moreover, it has also been reported that light intensity controls anti-predatory defences in *Daphnia*^29^.

The effects of the pharmacological treatments were consistent for GABAergic and muscarinic cholinergic compounds across two or three identical non-consecutive experiments performed over more than one year. Consistency of the toxicological results and, in particular, of the behavioural responses should be compulsory in toxicological studies to increase the credibility and robustness of the findings^30,31^. Agonists of these two neurotransmitter receptors (DZP, PILO) and the antagonist of the GABA receptor (PICRO) affected the induction of the phototactic behavioural changes (i.e., interfered with fish recognition). The receptor agonists DZP and PILO counteracted the negative phototactism evoked by the FKs, whereas PICRO enhanced the effect of the FKs, increasing the negative phototactism. None of the three applied substances when applied alone induced anti-predatory fish phototactic behaviour, indicating that these compounds interfered with the FK sensorial pathway. Alternatively, the muscarinic cholinergic antagonist SCOP interfered with phototaxis itself, almost completely abolishing the positive phototactic behaviour of the studied clone under both control and FK conditions. This indicates that the muscarinic cholinergic signalling pathway could potentially be a major regulator of anti-predatory fish phototactic behaviour. In *D. pulex* and *D. galeata*, the formation of neck teeth or helmets in response to predatory kairomones released by invertebrate predators has been related to a series of biological reactions that involve kairomone perception and neuronal signals, which are converted into endocrine signals and subsequently induce changes in the expression of morphogenetic factors^32,33^. We previously showed that DZP, PILO, PICRO and SCOP were neuroactive in *D. magna*, affecting sensitization and/or habituation motile responses to repetitive light stimuli^34^; thus, it is likely that these compounds disrupted neurological signalling pathways related to the phototactism shifts caused by FK perception or to the phototaxis itself.

Little is known about how phototaxis is neuronally coded. In *D. pulex,* both in silico and experimental works have shown that histaminergic neurons may mediate phototactic responses to UV irradiation^12^. By using histamine immunohistochemistry, the previous authors labelled putative photoreceptors in the compound eye and neuronal projections from these cells to the brain. The *D. pulex* genome also has a putative *Drosophila* orthologue of histidine decarboxylase (the rate-limiting biosynthetic enzyme for histamine), as well as two putative histamine-gated chloride channels (hclA and hclB orthologues). Exposure of *D. magna* to cimetidine, an H2 receptor antagonist known to block both hclA and hclB in *D. melanogaster*, inhibited the negative phototactic responses of these orthologues to UV irradiation. In another study, it was found that short-day photoperiods induced a significant increase in light-avoidance behaviours relative to controls and increased glutamate signalling, which is a critical pathway in arthropod light-avoidance behaviour^35^. It has also been reported that a group of serotonergic cells located in the protocerebrum probably control phototactic behaviour^16^. Notably, the perception of predatory kairomones and neuronal and cellular wiring is largely unknown in *Daphnia*^2^. For example, the receptors that detect invertebrate cues from *N*otonecta in *D. longicephala* were shown to be located on the first antennae, from which neurites extend into the deutocerebrum of the brain. However, key olfactory neuronal structures, such as olfactory glomeruli in the deutocerebrum, were not found^2^.

Our results obtained for DZP, an agonist of the GABA_A_ receptor, agree with those of Weiss et al.^11^, who found that co-exposure to FKs and exogenous GABA ameliorated life history changes to FKs in a *D. pulex* clone, whereas co-exposure with the GABA_A_ antagonist PICRO did not have any effect. The ineffectiveness of PICRO on the modulation of FK effects in *D. pulex* found by Weiss et al.^11^ might indicate species differences resulting from different receptor amino acid sequences. For example, GABA_A_ receptor subtypes with a single amino acid replacement make the *Drosophila* GABA_A_ receptor PICRO-insensitive^36^. Indeed, in crustaceans, lobster GABA_A_ receptors were also found to be insensitive to PICRO^37^. There is also the possibility that FK-mediated changes in phototactic behaviour and life history traits may be controlled by different mechanisms^6^.

Reported information on the modulatory effects of cholinergic compounds on anti-predatory defences in *Daphnia* is limited to invertebrate predatory cues, which, according to previous studies, should be regulated by neurological mechanisms distinct from those of fish^2,11^. Our results showed that the neurological cholinergic mechanisms that modulate induced defence responses against invertebrate predators or that mimic these responses are also able to do the same for fish predation but in the opposite way. Physostigmine and carbaryl, which are acetylcholinesterase inhibitors that increase acetylcholine receptor activity, enhanced and mimicked, respectively, the morphogenetic effects of invertebrate kairomones in several *Daphnia* species^11,21,23^. Conversely, atropine, which is a muscarinic acetylcholine receptor (AChR) inhibitor like SCOP, diminished neck tooth formation in *D. pulex*^11,21^. In our study, SCOP alone abolished the positive phototactism of the studied clone, which mimicked the effects of the FKs. Conversely, PILO, which is a muscarine AChR agonist, ameliorates the phototactic responses to FKs.

The nicotinic AChR agonists (NICO, IMI) and antagonist (MEC) only marginally affected the phototactic responses to the FKs. This indicates that muscarinic cholinergic signalling but not nicotinic signalling is involved in phototaxis/phototactic behaviour. It is therefore possible that both FK and SCOP treatment, through inhibition of muscarinic cholinesterase receptor activity, diminished the positive phototaxis of the studied clone, and PILO activation of these receptors ameliorated the effects of the FKs. In insects, neurons that connect olfactory inputs to higher-order brain areas that coordinate behavioural responses are thought to be under cholinergic control^38^.

In general, GABA is known to have inhibitory functions. It has been proposed that the continuous activation of the GABAergic neuronal pathway by endogenous GABA without predatory cues prevents life history shifts^11^, which in our case would be the transition from positive to negative phototaxis. FKs and PICRO relieve inhibition, which can be re-established by the experimental application of GABA_A_ receptor agonists such as DZP or GABA itself. Our results and those of Weiss et al.^11^ agree with the previous argument.

Equi-effective mixtures of the tested agonists and antagonists had similar effects on *D. magna* responses to FKs as the single mixture compound treatments did, indicating that the joint effects of agonists and antagonists of the GABAergic and cholinergic signalling pathways can act cooperatively and probably independently, modulating the effects of FKs. This is in line with other findings that showed that key ecophysiological responses in *Daphnia* are regulated by several signalling receptor pathways, which likely ensures more robust control. This is the case for the storage lipid dynamics associated with moulting and reproduction^39^.

The involvement of additional neurotransmitter signalling pathways, such as the serotonergic pathway, can also be taken into consideration despite being less consistent. Agonists of the serotonin receptor (such as serotonin) or treatments that increase serotonin levels (such as fluoxetine) ameliorated the effects of the FKs in only one experiment, but treatments that decreased serotonin, such as PCPA, increased the effects of the FKs in two out of the three experiments. Previously, we reported that serotonin activity in the brains of *D. magna* increased with algae food levels, and thus, the effects of fluoxetine on the enhancement of brain serotonin levels could only be observed under limited food conditions^24^. This indicates that the high levels of food used in our experiments probably prevented fluoxetine from increasing the already high serotonin levels in the central nervous system. Interestingly, inducible fish kairomone changes in phototactic behaviour in *Daphnia* increased with food level^40^, which is probably related to high levels of serotonin. On the other hand, the effects of PCPA, which decreases serotonin concentrations^26^, are unlikely to be modulated by food since this drug inhibits tryptophan hydrolase, the serotonin synthesis rate-limiting enzyme in *D. magna*^41^. This is apparently the case in our study.

Neurophysiological stimulation experiments with dopaminergic/adrenergic agonists and antagonists were inconclusive since in only one out of two experiments the dopaminergic agonist APO diminish negative phototaxis after FK exposure. We also did not find any effects from the glutamatergic agonists and antagonists on phototactism. This could be related to the low stability of dopaminergic compounds in water and the reported small effects of glutaminergic compounds on the *Daphnia* motile response to light^34^.Consistent failure of the tested antihistaminergic drugs to modulate phototactism to visible light disagrees with previous findings that discovered that these drugs affected phototactism but at much higher doses^12^.

### Metabolomic changes

The study of metabolomic changes across the treatments that modulated FK-mediated phototactic changes or altered phototaxis provided further experimental evidence of the involvement of key neurological signalling metabolic pathways. Caution must be exercised, however, since the studied receptor agonist and antagonist drugs do not change the neurotransmitters or their related metabolites. Nevertheless, little is known about how these drugs may affect the *Daphnia* neuronal metabolome. The cholinergic neurotransmitter system is one of the most important systems that plays a pivotal role in learning and memory in animal species, including *D. magna*^34,42^. Whole-body concentrations of acetylcholine decreased in females exposed to FKs and those exposed to SCOP and increased in those exposed to the agonists PILO and DZP. Thus, it is possible to establish a direct link between the decreased levels of acetylcholine and decreased positive phototactism in the studied clone. The results obtained for the GABAergic and serotonergic signalling pathways were less convincing, as FKs alone did not consistently affect the levels of GABA and serotonin. However, co-exposure to FK and the GABA_A_ receptor agonist DZP increased endogenous GABA levels, which is in line with the results reported by Weiss et al.^11^, who also found that the addition of exogenous GABA ameliorated FK effects. Interestingly, the summarized results depicted in Fig. 4 showed that serotonin levels dereased upon exposure to SCOP, PICRO and PILO but PILO also increase the levels of the serotonin degradation metabolite 5-HIAA. This may indicate that PILO may affect the turnover rather than the levls of serotionin.

Previous findings have reported altered responses to light in *D. magna* individuals lacking serotonin^16^. Therefore, it is possible to establish a link between the observed marked negative phototactism of females exposed to SCOP and low levels of serotonin.

Dopaminergic- and adrenergic-related metabolites deserve special attention, although there is only evidence that dopamine is involved in the proliferation and structural formation of morphological defences in *Daphnia* for invertebrate kairomones^22^. In some invertebrates, adrenergic signalling is considered to be absent, and the analogous functions are performed by octopamine^43^. In our study, fish kairomones and SCOP decreased the levels of dopamine and octopamine, whereas females co-treated with the agonists DZP and PILO and FKs showed relatively high levels of dopamine. In the insect *Drosophila melanogaster*, which shares many gene signalling pathways with Daphnia^44^, individuals deficient in dopamine show reduced positive phototactism^45^. Unfortunately, it is not possible to know whether the observed changes in DA in the whole bodies of *D. magna* indicate that DA is less used or used in excess. Figure 4 indicates that FK and SCOP reduced both DA and its intermediary metabolite L-DOPA. SCOP also increased the DA degradation metabolite 3-MT and two norepinephrine metabolites/neurotransmitters (NOEM, EPPY) that ultimately depend on DA. This means that FK decreased DA probably decreasing its intermediary metabolite L-DOPA, whereas SCOP decreased DA to a greater extent decreasing its intermediary L-DOPA but also increasing its turnover rate. Our neurophysiological stimulation experiments with dopaminergic active compounds are also not conclusive. This suggests that further research is needed to study the involvement of dopaminergic signalling in the response to fish. Existing studies on adrenergic signalling in daphnids indicated that β-blockers such as propranolol diminish the heart rate^46^ and motile responses to light^27^, which are related to the known role of adrenergic signalling that regulates blood pressure^47^ and other fight-or-flight responses to stress^48^. Future research is needed to elucidate the involvement of OCT, EPPY and NORM in the phototactic response of *D. magna* to FKs.

In summary, this study provides consistent results that muscarinic cholinergic and GABAergic receptor agonists and antagonists are able to ameliorate or enhance, respectively, the phototactic response of adult females from the studied *D. magna* clone to FKs. Furthermore, inhibition of the muscarinic acetylcholine receptor by SCOP induced the phototactic response to fish kairomones. This may indicate that muscarinic cholinergic antagonists changed phototaxis, whereas muscarinic cholinergic agonists and GABAergic agonists and antagonists changed the perception of FKs. Serotonergic agonists and antagonists were also able to diminish and increase FK effects, respectively, but only in half of the trials performed. The fact that we could not observe effects from the remaining neuroactive agents (i.e., dopaminergic, histaminergic, glutamatergic) could simply be because they are not relevant for predator-induced anti-phototaxis. The study of neurotransmitters and their related metabolite changes allowed us to identify acetylcholine and GABA as putative key metabolites associated with the observed phototactic modulatory effects of FK and cholinergic and GABAergic compounds. Increased and decreased levels of dopamine in the whole bodies of *D. magna* were related to positive and negative phototactic behaviours, respectively, but could not be related to neurophysiological studies with the tested dopaminergic drugs.

## Methods

### Experimental animals

The *D. magna* clone P_1_32,85 was obtained from two generations of intraclonal mixes within clone P_1_, which was isolated from a small pond that contained fish (Driehoeksvijver, Heusden; isolated in August 1986). Clone P_1_32,85 is known to become negatively phototactic in the presence of fish chemicals^5^. Bulk cultures of 10 animals/300 mL were maintained in ASTM hard water^49^ and fed every other day with 5 × 10^5^ cells/mL *Raphidocelis subcapitata*. Cultures were maintained until the adult females released their sixth brood and were then re-initiated with newborn individuals. To achieve the required number of experimental adult females needed for the behavioural tests, several larger cultures of 50 individuals/1.5 L were cultured and maintained for 15 days until use. These cultures were initiated with third- to sixth-brood neonates < 24 h old. The culture medium was renewed three times a week, the photoperiod was set to a 16 h light:8 h dark cycle, and the temperature was set to 20 ± 2 °C.

### Chemical compounds

Up to 16 modulators of the cholinergic, serotonergic, dopaminergic, histaminergic, GABAergic and glutamatergic neurotransmitter systems were tested (a brief description and the mode of action of each compound is provided in Supplementary Table S1). These compounds include nicotine (NICO), imidacloprid (IMI), mecamylamine (MECA) pilocarpine (PILO), scopolamine (SCOP), fluoxetine (FX), chloro-DL-phenylalanine (PCPA), serotonin (5-HT), 6-hydroxydopamine (6OH), apomorfine (APO), diphenhydramine (DIPH), cimetidine (CIM), diazepam (DZP), picrotoxin (PICRO), memantine (MEM) and N-methyl-D-aspartic acid (NMDA). The companies from which the salts or pure compounds were purchased are provided in the Supplementary Materials.

### Preparation of kairomone-enriched media

To obtain fish kairomone water-borne compounds (FKs), two 8 cm juvenile fish (*Leuciscus idus*) were allowed to swim in 15 L of ASTM hard water for 24 h, after which the water was filtered (0.045 µm), and several serial dilutions were examined to obtain moderate exposure levels. A final dilution of eight fold was used for this study. This procedure was repeated for each experiment. This method is routinely used to simulate fish predation risk^8,50^. The kairomone concentration corresponded roughly to 1 fish in 60 L.

### Experimental procedures

For each compound, only a single concentration was selected among those that had the greatest modulatory effect on the photomotor responses obtained in a previous study^34^. For certain compounds, however, the final concentrations were changed slightly following preliminary assays. The tested concentrations were 1 μg/L for PICRO, 100 μg/L for FX, SER, DZP, APO, SCOP, MECA, NICO and MEM and 1000 μg/L for IMI, PCPA, PILO, 6OH, DIPH, CIM and NMDA. Furthermore, the joint toxicity of the selected compounds was also tested using equi-effective binary mixtures, whose constituents were dosed at ½ of their single exposure concentration^51^. Each compound/treatment was tested at least twice in different experiments temporally separated by at least 1 month.

Except for DZP, whose stocks were prepared in ethanol, stocks for the rest of the compounds were prepared in Milli-Q water on the day of the experiment. The final concentration of ethanol in the DZP and control solvent solutions was 10 μL/L. Experimental treatments for each compound were defined as follows: control, FK (fish kairomone-conditioned water), compound alone or a mixture or combination of both treatments.

Chemical stability studies in ASTM water showed that most compounds were stable in water, except for 6-OH and APO, whose concentrations decreased over time^34^.

*D. magna* adult females (15 days old) were pre-exposed to the selected treatments for 24 h in groups of 5–6 individuals in 300 mL of test medium in 500 mL glass vessels prior to behavioural assays. Ten to fifteen individuals from two to three glass vessels, which was considered to be the lowest level of replication, was performed per treatment. The pre-exposure period was chosen considering that after 3 h of FK exposure, it is possible to detect changes in *Daphnia* behaviour^8^. Pre-exposures were conducted with food (5 × 10^5^ cells/mL *R. subcapitata).*

### Swimming behaviour assay

Following exposure, the swimming tracks of 15-d-old females from clone P_1_32,85 pre-exposed for 24 h to the studied treatment were assessed using a custom-designed experimental chamber containing two independent arenas (8 × 4 × 2 cm, H × W × D) with backlight infrared illumination. The apically located LED stripe producing visible white light and the GigE camera located in front of the arenas were controlled by EthoVision XT 11.5 software (Noldus Information Technology, Leesburg, VA). The bottoms of the arenas were black to minimize light scattering and reflection. Further details of the behavioural device are provided elsewhere^27^. For each compound and FK combination, several behavioural trials were performed, depending on the number of glass vessels containing the groups of experimental individuals used. In each trial, groups of 5 or 6 *Daphnia* from two different treatments were distributed among the two arenas filled with 50 mL of test solution without food. Thus, a total of 10–15 individuals/replicates were monitored per treatment. Treatments were randomized across chambers. Animals were then acclimated in the dark for 10 min before video recording. For behavioural analysis, the animals were recorded in the dark (5 min) and under moderate-intensity apical white light (375 lx, 15 min). The use of lower (96 lx) and higher (1154 lx) light intensities provided less consistent results (see Supplementary Material for further details). After video recording at 20 frames per second (fps), the EthoVision XT 14 video-tracking software was used to analyse the changes in the position of each animal. First, each arena was divided into three identical virtual zones, corresponding to the top, middle and bottom. Then, the individual tracks of the five or six experimental animals in each arena were analysed by using the social interaction module of the software, which determined the time spent in the top virtual zone (%). For statistical analysis for each individual, the mean value of the last ten minutes of the light period was considered. Further information regarding method optimization and validation is provided in the Supplementary Material. Values were determined per minute.

### Metabolomic study

Five to ten replicates of selected treatments (DZP, PILO, PICRO, SCOP) and their respective controls were used for metabolomics analyses. Two separate experiments were performed. Experiment 1 included a control, PILO, PICRO and SCOP treatment both alone and in combination with FKs, whereas experiment 2 included an ethanol dilution control, DZP alone and DZP with FKs. Exposure conditions were identical to those used in the behavioural assays. Following exposure, animals were sampled and pooled in groups of five in an Eppendorf tube, the water was removed, and the samples were deep frozen in liquid N_2_. Samples were stored at − 80 °C until analysis. Metabolites were analysed by liquid chromatography coupled with tandem mass spectrometry following the procedures of previous studies^25,26,52^ with minor modifications. Briefly, metabolites were extracted with acetonitrile acidified with formic acid and with the addition of the antioxidant ascorbic acid (further details are in the Supplementary Material).

### Data analyses

The behavioural experimental design followed a two-way nested mixed ANOVA design with FK and compound treatments used as fixed factors and the two or three glass vessels/arenas used per treatment as a nested random factor. The number of individual replicates per treatment, which varied between 10 and 15, was used as the lowest replicate level. Metabolomic responses were compared by a two-way ANOVA design with FK and compound treatments as fixed factors. Prior to analyses, the percentage or metabolite concentration data were tested to meet ANOVA assumptions of normality and variance homoscedasticity and if needed, it was arccosine- (for %) or log-transformed. Following ANOVAs, differences among the treatments were further compared using Tukey’s post hoc multiple comparisons test. When data did not meet the ANOVA assumptions of normality and variance homoscedasticity even after transformation, a non-parametric one-way ANOVA Kruskal–Wallis test followed by a non-parametric equivalent Tukey’s test were used^53^. Analyses were performed with IBM SPSS Statistics software v27.

## Supplementary Information

Supplementary Information.

## Data Availability

The datasets generated during and/or analysed during the current study are available from the corresponding author on reasonable request.
