# Supplementary material for: Pharmacological modulation of fish-induced depth selection in D. magna: the role of cholinergic and GABAergic signalling
Source: Sci Rep. 2021 Sep 30;11:19407. doi: 10.1038/s41598-021-98886-w (PMC8484359; doi:10.1038/s41598-021-98886-w)
Supplement: Supplementary file 1 — Supplementary Information. [file 41598_2021_98886_MOESM1_ESM.docx]

**Supplementary Material**

**Title**: Pharmacological modulation of fish-induced depth selection in *D. magna*: The role of cholinergic and GABAergic signalling

Juliette Bedrossiantz^1,^ , Inmaculada Fuertes^1^, Demetrio Raldua^1^, Carlos Barata^1^

**Author affiliations:**

^1^Department of Environmental Chemistry, Institute of Environmental Assessment and Water Research, CSIC, Jordi Girobna 18, 08034 Barcelona, Spain

**Correspondence to**: Carlos Barata, [cbmqam@cid.csic.es](mailto:cbmqam@cid.csic.es)

**METHODS**

**Chemical compounds**

The following compounds were purchased from Sigma-Aldrich (St. Louis, MO): nicotine; pilocarpine hydrochloride; scopolamine hydrochloride fluoxetine hydrochloride; chloro-DL-phenylalanine; serotonin creatinine sulfate monohydrate, imidacloprid, 6-hydroxydopamine hydrochloride, apomorfine hydrochloride, diphenhydramine hydrochloride, cimetidine, diazepam, picrotoxin, memantine hydrochloride and N-Methyl-D- aspartic acid. Mecamylamine hydrochloride was purchased from Tocris Bioscience (Minneapolis, MN).

**Method optimization**

It aimed to select an optimal fish kairomone concentration, light intensity and photoperiod to maximize the detection of FK effects on Daphnia phototaxis. Accordingly animals were initially exposed for 24 h to filtered conditioned ASTM water of three fish stocking densities, 1 fish/120 L, 1 fish/60 L, 1 fish/30 L. Treatments were tested twice across experiments temporally separated at least 1 month.

For the behavioral analysis, animals were recorded in the dark (5 min), and under three apical white light intensities (96, 375 and 1154 lux for, 15 min). After video-recording the changes in the position of each animal were analyzed. First of all, each arena was divided in three identical virtual zones, corresponding with the top, middle and the bottom of the arena. Then, individual tracks of the five or six experimental animals in each arena were analyzed by using the social interaction module of the software, determining the total distance moved (cm) and time spent in the top, middle and bottom virtual zone (%). For statistical analyses for each individual the mean value of three periods was considered: the last five minutes of the dark period, the first five and the last ten minutes of light period, hereafter referred as dark (dk), first (L1) and late light (L2), respectively.

**Metabolome analysis**

*Sample extraction*

Samples were extracted following previous studies ^1^, with minor modifications ^2^. In brief, 500 µL of ACN with 0.1% formic acid and 0.02% ascorbic acid were added to samples each corresponding to pools of five *D. magna* individuals. Samples were spiked with 50 ng/mL of isotope labeled solution and were shaken. Following that, samples were homogenized using a bead mill homogenizer (TissueLyser LT, Qiagen), at 50 oscillations per min during 60 seconds. Subsequently, samples were centrifuged at 10 000g during 10 min at 4ºC. The supernatant was transferred to a new tube and left in ice during 30 minutes in order to precipitate proteins. Samples were centrifuged again, and the supernatant was transferred to a new tube and evaporated to complete dryness under mild nitrogen, when samples were kept at -80ºC until analysis. On the day of the analysis, samples were re-suspended in 60 µL of ACN:H_2_O (50:50, v/v), centrifuged again at 10000g during 10 min at 4ºC, and transferred to an insert amber glass chromatographic vial for its analysis.

*UPLC-MS/MS analysis*

Targeted neurotransmitters were measured using Ultra Performance Liquid Chromatography couple to tandem mass spectrometry (UPLC-MS/MS), consisting of an Acquity UPLC system (Waters, USA) connected to a Xevo triple quadrupole mass spectrometer (Waters, USA), as described in Fuertes et al. ^3^ Neurotransmitters were measured under positive electrospray ionization (ESI+). Mass spectrometer conditions were those reported in Fuertes et al.^3^ Experimental data were acquired and processed using the MassLynx v4.1 software package (Waters, USA).

Sample quantification of all the targeted neurotransmitters was performed by external standard calibration, prepared into *Daphnia magna* neurotransmitter samples to account for matrix effects and corrected by one of the internal standard labelled compounds to correct extraction efficiencies and analytes mass spectrometer responses, ensuring exact quantification performance. Table S2 displays detailed information on the internal standard used for each compound, as well as the quality parameters that were determined, as linearity (determined by the regression coefficient over the calibration range), recovery, instrumental detection limit (IDL), method detection limit (MDL) and method quantification limit (MQL).

**RESULTS**

**Method optimization**

The proposed image video analysis consisting in a vertical two arena set up with an apical white light LED source was able to monitor the phototactic trajectories of the females adults of the studied clone (Fig S1). Adults of the tested clone were located predominantly in the top of the arena during the dark period (Dk). During the first light period (L1), and in particular during the first minute after the sudden turn on of the apical illumination, some of the individual move from the top to the bottom. Finally, during the second light period (L2) individuals move again towards the top (L2) (Fig S1). This behavior was consistent across the two experiments depicted in Fig S1 (A, B). The rest of graphs (Fig S1C-H) show that FK increases the number of individual moving to the bottom during L1 period and also reduces the return of the individuals to the top commonly observed during L2 period in individuals of this clone without FK.

**Fig S1. Individuals from clone P_1_32,85 have a marked positive phototactic time course response that became negative upon FK**. Percentage of individuals swimming in the upper, middle and bottom virtual zones of the experimental arena (Mean ± SE, N =15) of *D. magna* females across the studied dark and light periods and FK dilution water treatments. Right and left panel graphs show data from two experiments. Treatments included, controls (A,B), 1 fish/120 L (C,D), 1 fish/60 L (E, F), 1 fish/30 L (G;H).

The above mentioned phototactic trajectories were summarized and compared statistically in Fig 2 (A, B), which shows the % of individuals swimming in the top virtual zone during the dark period (Dk), the first (L1) and the last (L2) light periods. ANOVA results depicted in Table S3, indicated that FK decreased significantly (P<0.05) positive phototactism during the whole light period, but specially during the late light period (L2). Phototactic behavior was tightly regulated by light intensity being the response to fish kairomones more marked at 375 than at 96 lux. Higher light intensities (1154 lux) decreased the positive phototactism of the studied clone and also its response to FK (Fig S2C).

**Fig S2.** **Phototactic responses to FK were more apparent during the late period of light and at intermediate light intensity of 375 lux, and increased with the stocking density of fish**. The Percentage of individuals swimming in the top virtual zone (Mean ±SE, N =10-15) across the studied photoperiods (Dark, Dk; first,L1, and late period of light,L2), FK dilution treatments (A, B) and light intensities (C). Fish densities of 30, 60 and 120 are respectively 1 fish in 30, 60 and 120 L. A and B report results from experiments also depicted in Fig S1. Different letters means significant (P<0.05) differences following parametric or non parametric ANOVA and multiple compassion tests.

The total distance moved by *D. magna* individuals across the dark and light periods and FK treatments was not consistent across experiments (Fig S3), thus phototactic behavior responses across the studied compounds were limited to analyze the % of individuals located in the most distinctive virtual zone (the top one).

**Fig S3. The change in locomotor activity was not consistent across FK treatments.** Trajectories of the total distance moved (Mean ± SE, N=15) of *D. magna* females within the entire arena across the studied dark and light periods and FK dilution water treatments (A, B). The mean distance moved across periods (Dark, Dk; first,L1, and late period of light, L2) are depicted in graphs C, D. Different letters means significant (P<0.05) differences following ANOVA and multiple compassion tests.

**Tables**

**Table S1**. **Up to 16 compounds known to modulate six neurological signalling pathways were tested**. Description and putative mode of action.

**Table S2 Quality parameters obtained by LC-MS/MS for the targeted compounds**. F: slope; R^2^: regression coefficient; IS, internal standard; IDL: instrumental detection limit; MDL: method detection limit; MQL: method quantification limit.

| **Target Compound** | **Labelled IS** | **Range** | **F** | **R²** | **Recovery** | | | **IDL** | **MDL** | **MDQ** |
| --- | --- | --- | --- | --- | --- | --- | --- | --- | --- | --- |
|  |  | **(ng/mL)** |  |  | **(mean ± SD, %)** | | | **(pg)** | **(pg/daphnia)** | **(pg/daphnia)** |
| **3-MT** | 3-MT-d4 | 5-500 | 0,936 | 0,9991 | 57,46 | ± | 1,0 | 1,05 | 1,198 | 3,992 |
| **5-HIAA** | 5-HIAA-d5 | 5-500 | 15,312 | 0,9731 | 87,54 | ± | 6,1 | 4,08 | 0,00361 | 0,01203 |
| **5-HTP** | 5-HTP-d4 | 5-500 | 2,079 | 0,9981 | 71,11 | ± | 2,5 | 6,25 | 0,00187 | 0,00624 |
| **Acetylcholine (ACH)** | Acetylcholine-d9 | 5-500 | 0,330 | 0,9992 | 55,00 | ± | 4,0 | 2,31 | 5,61 | 18,71 |
| **Choline (CHO)** | Choline-13C | 10-500 | 2,547 | 0,9727 | 123,22 | ± | 9,5 | 0,09 | 1,29 | 4,291 |
| **Dopamine (DA)** | Dopamine-d4 | 5-500 | 4,045 | 0,9879 | 49,33 | ± | 1,2 | 0,55 | 1,20 | 3,985 |
| **Epinephrine (EPPY)** | Epinephrine-d6 | 5-500 | 0,937 | 0,9998 | 57,52 | ± | 1,7 | 0,89 | 0,361 | 1,203 |
| **GABA** | GABA-d6 | 5-500 | 1,889 | 0,9922 | 44,92 | ± | 7,0 | 0,12 | 12,9 | 42,96 |
| **Histamine (HSM)** | Histamine-d4 | 5-500 | 1,139 | 0,9908 | 63,72 | ± | 3,6 | 2,37 | 7,26 | 24,21 |
| **L-DOPA** | L-DOPA-d3 | 5-500 | 1,789 | 0,9965 | 37,05 | ± | 3,1 | 1,60 | 0,00551 | 0,01835 |
| **Norepinephrine (NOR)** | Norepinephrine-d6 | 5-500 | 1,700 | 0,9977 | 55,87 | ± | 1,5 | 0,99 | 0,302 | 1,006 |
| **Normetanephrine (NORM)** | Dopamine-d4 | 10-500 | 0,041 | 0,9594 | 48,89 | ± | 5,6 | 0,10 | 2,18 | 7,266 |
| **Octopamine (OCT)** | Dopamine-d4 | 5-500 | 0,363 | 0,9858 | 61,74 | ± | 3,7 | 1,79 | 7,03 | 23,42 |
| **Phenylalanine (PHE)** | Phenylalanine-13C | 10-500 | 1,001 | 0,9527 | 49,88 | ± | 11,7 | 0,54 | 2,60 | 8,675 |
| **Serotonin (5-HT)** | Serotonin-d4 | 5-500 | 4,652 | 0,9860 | 51,87 | ± | 5,9 | 0,21 | 0,304 | 1,014 |
| **Taurine (TAU)** | Taurine-15N | 5-500 | 1,071 | 0,9983 | 51,27 | ± | 5,2 | 6,43 | 70,2 | 234,0 |
| **Tryptophan (TRP)** | Tryptophan-13C | 5-500 | 0,870 | 0,9749 | 49,21 | ± | 3,2 | 0,43 | 3,51 | 11,71 |

*GABA* γ-aminobutyric acid, *5-HTP* 5-hydroxy-tryptophan, *L-DOPA* 3,4-dihydroxyphenylalanine, *5-HIAA* 5-hydroxyindoleacetic acid, *3-MT* 3-methoxytyramine

**Table S3. Statistical results for method optimization**. Include those for the two FK dilution experiments (Exp 1, Exp2) and the one testing light intensity. KW. Non parametric Kruskal-Wallis; Nested ANOVA with Arena nested across FK dilution or light intensities Dk, L1, L2 are, respectively, the dark, first and last light periods. % and D are % of individuals and the distance moved within each period, respectively. df, and P are respectively, degrees of freedom and P values.

| KW |  |  |  |  |  |  |
| --- | --- | --- | --- | --- | --- | --- |
| Fish kairiome water Dilution experiments | | | |  |  |  |
| Exp 1 | df | KW | P |  |  |  |
| %DK | 3 | 2.0 | 0.572 |  |  |  |
| %L1 | 3 | 19.3 | <0.001 |  |  |  |
| %L2 | 3 | 32.4 | <0.001 |  |  |  |
| Exp 2 |  |  |  |  |  |  |
| %DK | 3 | 1.1 | 0.78 |  |  |  |
| %L1 | 3 | 27.1 | <0.001 |  |  |  |
| %L2 | 3 | 35.4 | <0.001 |  |  |  |
| Nested ANOVA | |  |  |  |  |  |
| Exp 1 | Arena |  |  | FK Dilution | |  |
|  | df | F | P | df | F | P |
| DDk | 4,32 | 0.7 | 0.576 | 3,4 | 1 | 0.488 |
| DL1 | 4,32 | 0.3 | 0.87 | 3,4 | 2.6 | 0.193 |
| DL2 | 4,32 | 2.1 | 0.107 | 3,4 | 6.1 | 0.056 |
| Exp 2 |  |  |  |  |  |  |
| DDk | 8,46 | 2 | 0.065 | 3,8 | 0.3 | 0.821 |
| DL1 | 8,48 | 1.4 | 0.232 | 3,8 | 3.9 | 0.055 |
| DL2 | 8,48 | 0.2 | 0.99 | 3,8 | 28.9 | <0.001 |
| Light Intensity Experiment | | |  |  |  |  |
|  | tank |  |  | Light Intensity | |  |
|  | df | F | P | df | F | P |
| %L2 | 6,48 | 1 | 0.453 | 5,6 | 15.44 | 0.002 |

**Table S4. Non parametric statistical results for the phototactic effects of the studied pharmaceuticals and FK.**  Kruskal-Wallis results for the % of individuals swimming on top during the late light period across the tested compounds and experiments. Experiment replicate numbers (from 1 to 3) are depicted after compound abbreviations. df, KW and P are respectively, degrees of freedom, Kruskal-Wallis test and P-values.

|  | df | KW | P |
| --- | --- | --- | --- |
| DZP 1 | 3 | 17.2 | 0.001 |
| DZP 3 | 3 | 41.7 | <0.001 |
| PICRO 2 | 3 | 35.0 | <0.001 |
| SCOP 1 | 3 | 28.5 | <0.001 |
| SCOP2 | 3 | 36.7 | <0.001 |
| SCOP 3 | 3 | 47.4 | <0.001 |
| APO1 | 3 | 25.0 | <0.001 |
| 6OH 1 | 3 | 32.0 | <0.001 |
| 6OH 2 | 3 | 17.8 | <0.001 |
| DIPH 1 | 3 | 36.7 | <0.001 |
| DIPH 2 | 3 | 16.3 | 0.001 |
| CIM 1 | 3 | 35.9 | <0.001 |
| CIM 2 | 3 | 24.8 | <0.001 |
| FX 1 | 3 | 41.0 | <0.001 |
| FX 2 | 3 | 11.4 | 0.01 |
| 5HT 1 | 3 | 39.9 | <0.001 |
| 5HT 2 | 3 | 21.4 | <0.001 |
| PCPA 1 | 3 | 48.1 | <0.001 |
| PCPA 2 | 3 | 29.2 | <0.001 |
| IMI 1 | 3 | 26.4 | <0.001 |
| Mix ANT 1 | 3 | 29.3 | <0.001 |
| Mix AG 1 | 3 | 23.2 | <0.001 |
| Mix ANT 2 | 3 | 26.8 | <0.001 |

**Table S5. Two-way nested ANOVA results for the phototactic effects of the studied compounds on FK.** It tested the effect of arena (nested factor) and of FK and compound on the % of individuals swimming on top during the late light period across the tested compounds and experiments. Experiment replicate numbers (from 1 to 3) are depicted after compound abbreviations. df, F and P are respectively, degrees of freedom, Fisher’s coefficient and P-values.

|  |  | Arena |  |  | FK |  |  | Drug |  |  | Interaction | |  |
| --- | --- | --- | --- | --- | --- | --- | --- | --- | --- | --- | --- | --- | --- |
|  |  | df | F | P | df | F | P | df | F | P | df | F | P |
| DZP 2 | L2 | 4,32 | 0.4 | 0.781 | 1,4 | 26.4 | 0.007 | 1,4 | 17.9 | 0.013 | 1,4 | 0.0 | 0.947 |
| PICRO 1 | L2 | 4,32 | 0.3 | 0.860 | 1,4 | 95.5 | 0.001 | 1,4 | 34.4 | 0.004 | 1,4 | 8.2 | 0.046 |
| PILO 1 | L2 | 9,52 | 1.0 | 0.490 | 1,9 | 16.3 | 0.003 | 1,9 | 8.7 | 0.016 | 1,9 | 6.5 | 0.031 |
| PILO 2 | L2 | 4,32 | 0.4 | 0.836 | 1,4 | 30.8 | 0.005 | 1,4 | 13.4 | 0.022 | 1,4 | 52.6 | 0.002 |
| PILO 3 | L2 | 4,32 | 0.7 | 0.575 | 1,4 | 11.3 | 0.028 | 1,4 | 0.0 | 0.907 | 1,4 | 18.2 | 0.013 |
| NICO 1 | L2 | 10,51 | 1.7 | 0.117 | 1,10 | 13.5 | 0.003 | 1,10 | 4.0 | 0.069 | 1,10 | 2.3 | 0.157 |
| NICO 2 | L2 | 4,31 | 2.0 | 0.123 | 1,4 | 28.5 | 0.006 | 1,4 | 0.4 | 0.568 | 1,4 | 1.2 | 0.331 |
| IMI 2 | L2 | 9,52 | 0.8 | 0.652 | 1,9 | 69.0 | <0.001 | 1,9 | 8.8 | 0.016 | 1,9 | 2.4 | 0.153 |
| MEC 1 | L2 | 9,50 | 1.2 | 0.292 | 1,9 | 47.2 | <0.001 | 1,9 | 0.8 | 0.405 | 1,9 | 2.4 | 0.154 |
| MEC 2 | L2 | 4,32 | 0.3 | 0.892 | 1,4 | 130.9 | <0.001 | 1,4 | 12.5 | 0.024 | 1,4 | 12.6 | 0.024 |
| APO 2 | L2 | 4,32 | 0.3 | 0.906 | 1,4 | 43.3 | 0.003 | 1,4 | 1.5 | 0.287 | 1,4 | 6.5 | 0.063 |
| FX 3 | L2 | 7,44 | 1.2 | 0.301 | 1,7 | 234.7 | <0.001 | 1,7 | 5.7 | 0.049 | 1,7 | 1.3 | 0.296 |
| 5HT3 | L2 | 7,44 | 0.4 | 0.890 | 1,7 | 574.9 | <0.001 | 1,7 | 109.8 | <0.001 | 1,7 | 28.0 | 0.001 |
| PCPA 3 | L2 | 7,44 | 0.4 | 0.886 | 1,7 | 562.9 | <0.001 | 1,7 | 22.7 | 0.002 | 1,7 | 50.3 | 0.000 |
| MEM 1 | L2 | 8,48 | 1.5 | 0.192 | 1,8 | 290.3 | <0.001 | 1,8 | 2.8 | 0.13 | 1,8 | 5.4 | 0.049 |
| MEM 2 | L2 | 8,48 | 2.0 | 0.066 | 1,8 | 35.1 | <0.001 | 1,8 | 1.3 | 0.286 | 1,8 | 0.1 | 0.728 |
| NMDA 1 | L2 | 6,40 | 2.0 | 0.092 | 1,6 | 293.1 | <0.001 | 1,6 | <0.001 | 0.869 | 1,6 | <0.001 | 0.914 |
| NMDA 2 | L2 | 8,48 | 1.2 | 0.330 | 1,8 | 76.0 | <0.001 | 1,8 | 5.0 | 0.057 | 1,8 | 2.5 | 0.154 |
| Mix AG 2 | L2 | 4,32 | 1.6 | 0.211 | 1,4 | 114.9 | <0.001 | 1,4 | 97.8 | 0.001 | 1,4 | 73.2 | 0.001 |

**Table S6. Two-way ANOVA testing the effects of FK and compound on the concentration of the studied metabolites** measured in whole *D. magna* tissues. df, F and P are respectively, degrees of freedom, Fisher’s coefficient and P-values.

|  | FK |  |  | Drug |  |  | Interaction |  |  |
| --- | --- | --- | --- | --- | --- | --- | --- | --- | --- |
| all but DZP | df | F | P | df | F | P | df | F | P |
| TAU | 1,42 | 0.3 | 0.615 | 3,42 | 0.9 | 0.434 | 3,42 | 0.4 | 0.744 |
| CHO | 1,42 | 3 | 0.093 | 3,42 | 1.7 | 0.176 | 3,42 | 0.5 | 0.698 |
| PHE | 1,42 | 1.5 | 0.235 | 3,42 | 0.6 | 0.616 | 3,42 | 0.8 | 0.523 |
| GABA | 1,42 | 4.7 | 0.036 | 3,42 | 19.7 | <0.001 | 3,42 | 1.2 | 0.339 |
| 5HTP | 1,42 | <0.1 | 0.968 | 3,42 | 6.2 | 0.001 | 3,42 | 0.9 | 0.453 |
| TRP | 1,42 | 0.3 | 0.613 | 3,42 | 10.1 | <0.001 | 3,42 | 0.2 | 0.924 |
| L-DOPA | 1,42 | 1.1 | 0.307 | 3,42 | 11.8 | <0.001 | 3,42 | 9.4 | <0.001 |
| 5-HIAA | 1,42 | 4.1 | 0.048 | 3,42 | 4.3 | 0.01 | 3,42 | 1 | 0.384 |
| EPPY | 1,42 | <0.1 | 0.843 | 3,42 | 4.4 | 0.009 | 3,42 | 1.5 | 0.232 |
| 3-MT | 1,42 | 0.1 | 0.714 | 3,42 | 28.1 | <0.001 | 3,42 | 2 | 0.129 |
| NORM | 1,42 | 0.2 | 0.694 | 3,42 | 4.9 | 0.005 | 3,42 | 1.5 | 0.236 |
| 5-HT | 1,42 | 0.2 | 0.671 | 3,42 | 15.5 | <0.001 | 3,42 | 0.9 | 0.471 |
| NOREP | 1,42 | 2.7 | 0.11 | 3,42 | 0.5 | 0.686 | 3,42 | 0.9 | 0.446 |
| ACH | 1,42 | 12.6 | 0.001 | 3,42 | 7 | 0.001 | 3,42 | 6.3 | 0.001 |
| DA | 1,42 | 3 | 0.091 | 3,42 | 4.1 | 0.013 | 3,42 | 4.3 | 0.009 |
| HSM | 1,42 | <0.1 | 0.928 | 3,42 | 0.7 | 0.534 | 3,42 | 1.2 | 0.314 |
| OCP | 1,42 | 4.7 | 0.036 | 3,42 | 22.5 | <0.001 | 3,42 | 1.2 | 0.339 |
| DZP | df | F | P | df | F | P | df | F | P |
| TAU | 1,16 | <0.1 | 0.954 | 1,16 | 0.2 | 0.667 | 1,16 | 0.1 | 0.807 |
| CHO | 1,16 | <0.1 | 0.902 | 1,16 | 0.2 | 0.635 | 1,16 | 0.1 | 0.761 |
| PHE | 1,16 | 0.1 | 0.777 | 1,16 | 0.6 | 0.462 | 1,16 | 0.3 | 0.619 |
| GABA | 1,16 | 3.9 | 0.065 | 1,16 | 0.5 | 0.48 | 1,16 | 7.2 | 0.016 |
| 5-HTP | 1,16 | 0.1 | 0.815 | 1,16 | 1.8 | 0.204 | 1,16 | <0.1 | 0.854 |
| TRP | 1,16 | 0.4 | 0.517 | 1,16 | 0.1 | 0.721 | 1,16 | <0.1 | 0.95 |
| L-DOPA | 1,16 | 13.5 | 0.002 | 1,16 | 152.8 | <0.001 | 1,16 | 10.9 | 0.005 |
| 5-HIAA | 1,16 | 70.9 | <0.001 | 1,16 | 86.1 | <0.001 | 1,16 | 10.5 | 0.005 |
| EPPY | 1,16 | 7.5 | 0.014 | 1,16 | 26.8 | <0.001 | 1,16 | 8.2 | 0.011 |
| 3-MT | 1,16 | 0.8 | 0.393 | 1,16 | 1.3 | 0.273 | 1,16 | 0.1 | 0.738 |
| NORM | 1,16 | 0.3 | 0.617 | 1,16 | 1.9 | 0.182 | 1,16 | 1.7 | 0.215 |
| 5-HT | 1,16 | 0.3 | 0.615 | 1,16 | 0.1 | 0.708 | 1,16 | 0.5 | 0.477 |
| NOREP | 1,16 | 0.2 | 0.646 | 1,16 | 2.2 | 0.161 | 1,16 | 1.9 | 0.184 |
| ACH | 1,16 | 0.2 | 0.657 | 1,16 | 10.7 | 0.005 | 1,16 | 1.1 | 0.317 |
| DA | 1,16 | 0.6 | 0.436 | 1,16 | 1.2 | 0.292 | 1,16 | 0.5 | 0.495 |
| HSM | 1,16 | 0.5 | 0.509 | 1,16 | 1.3 | 0.268 | 1,16 | 1.5 | 0.243 |
| OCP | 1,16 | 1 | 0.323 | 1,16 | 0.2 | 0.666 | 1,16 | 12.1 | 0.003 |

**Figures**

**Fig S4.** **Agonists and antagonists of the muscarinic acetylcholine and GABA_A_ receptors consistently affected the phototactic time course response of adult females to FK.** Percentage of individuals swimming in the upper zone (Mean ± SE, N =10-15) of *D. magna* females across the studied dark and light periods following exposure to FK, DZP, PICRO, PILO. Results from two or three experiments are depicted. For clarity number of minutes (1-20) are not depicted in the x axis. C, Comp, FK, Comp+FK are, respectively, control, compound, fish kairomone and co-exposures of compound and fish kairomone treatments.

**Fig S5. Mixtures of agonists and antagonists of the muscarinic acetylcholine and GABA_A_ receptors consistently affected the phototactic time-course responses of adult females to FK.** Percentage of individuals swimming in the upper zone (Mean ± SE, N =10-15) of *D. magna* females across the studied dark and light periods following exposure to FK and binary mixtures of antagonists (A, B) and agonists (C,D). Results from two experiments are depicted. For clarity number of minutes (1-20) are not depicted in the x axis. Legend abbreviations are explained in Fig S4.

**Fig S6.** **Inhibition and stimulation of the nicotinic acetyl cholinergic, dopaminergic and histaminergic signaling pathways only marginally modulate phototactic time course responses to FK.**  Percentage of individuals swimming in the upper zone (Mean ±SE, N =10-15) of *D. magna* females across the studied dark and light periods following exposure to FK, IMI, NICO, MECA, APO, 6OH, DIPH. Results from two experiments are depicted. For clarity number of minutes (1-20) are not depicted in the x axis. Legend abbreviations are explained in Fig S4.

**Fig S7.** **Inhibition and stimulation of the histaminergic and serotonergic signaling pathways only marginally modulate phototactic time course responses to FK.**  Percentage of individuals swimming in the upper zone (Mean ± SE, N =10-15) of *D. magna* females across the studied dark and light periods following exposure to FK, CIM, SER, FX, PCPA. Results from two or three experiments are depicted. For clarity number of minutes (1-20) are not depicted in the x axis. Legend abbreviations are explained in Fig S4

Fig S8. **Inhibition and stimulation of the glutamatergic signaling pathway only marginally modulate phototactic time course responses to FK.**  Percentage of individuals swimming in the upper zone (Mean ± SE, N =10-15) of *D. magna* females across the studied dark and light periods following exposure to FK, MEM and NMDA. Results from two experiments are depicted. For clarity number of minutes (1-20) are not depicted in the x axis. Legend abbreviations are explained in Fig S4

**References**

1. Rivetti, C., Climent, E., Gómez-Canela, C. & Barata, C. Characterization of neurotransmitter profiles in *Daphnia magna* juveniles exposed to environmental concentrations of antidepressants and anxiolytic and antihypertensive drugs using liquid chromatography–tandem mass spectrometry. *Anal. Bioanal. Chem.* **411**, 5867–5876 (2019).

2. Fuertes, I. & Barata, C. Characterization of neurotransmitters and related metabolites in *Daphnia magna* juveniles deficient in serotonin and exposed to neuroactive chemicals that affect its behavior: A targeted LC-MS/MS method. *Chemosphere* **263**, 127814 (2021).

3. Fuertes, I. & Barata, C. Characterization of neurotransmitters and related metabolites in *Daphnia magna* juveniles deficient in serotonin and exposed to neuroactive chemicals that affect its behavior: A targeted LC-MS/MS method. *Chemosphere* **263**, (2021).

4. McCoole, M. D., Baer, K. N. & Christie, A. E. Histaminergic signaling in the central nervous system of *Daphnia* and a role for it in the control of phototactic behavior. *J. Exp. Biol.* **214**, 1773–1782 (2011).

5. Bedrossiantz, J. *et al.* A high-throughput assay for screening environmental pollutants and drugs impairing predator avoidance in *Daphnia magna*. *Sci. Total Environ.* **740**, (2020).

6. Miyakawa, H., Sato, M., Colbourne, J. K. & Iguchi, T. Ionotropic glutamate receptors mediate inducible defense in the water flea *Daphnia pulex*. *PLoS One* **10**, (2015).

7. Weiss, L. C., Leese, F., Laforsch, C. & Tollrian, R. Dopamine is a key regulator in the signalling pathway underlying predatorinduced defences in *Daphnia*. *Proc. R. Soc. B Biol. Sci.* **282**, (2015).

8. Bauknecht, P. & Jékely, G. Ancient coexistence of norepinephrine, tyramine, and octopamine signaling in bilaterians. *BMC Biol.* **15**, 6 (2017).

9. Adamo, S. A. Norepinephrine and octopamine: Linking stress and immune function across phyla. *Invertebr. Surviv. J.* **5**, 12–19 (2008).

10. Gallo, V. P., Accordi, F., Chimenti, C., Civinini, A. & Crivellato, E. Catecholaminergic System of Invertebrates: Comparative and Evolutionary Aspects in Comparison With the Octopaminergic System. in 363–394 (2016). doi:10.1016/bs.ircmb.2015.12.006.

11. Colbourne, J. K. *et al.* The ecoresponsive genome of *Daphnia pulex*. *Science (80-. ).* **331**, 555–561 (2011).

12. Riemensperger, T. *et al.* Behavioral consequences of dopamine deficiency in the Drosophila central nervous system. *Proc Natl Acad Sci U S A* **108**, 834–839 (2011).

13. Jeong, T. Y., Yoon, D., Kim, S., Kim, H. Y. & Kim, S. D. Mode of action characterization for adverse effect of propranolol in *Daphnia magna* based on behavior and physiology monitoring and metabolite profiling. *Environ. Pollut.* **233**, 99–108 (2018).

14. Simão, F. C. P. *et al.* Using a new high-throughput video-tracking platform to assess behavioural changes in *Daphnia magn*a exposed to neuro-active drugs. *Sci. Total Environ.* **662**, (2019).

15. Falkner, B. *et al.* The fourth report on the diagnosis, evaluation, and treatment of high blood pressure in children and adolescents. *Pediatrics* **114**, 555–576 (2004).

16. Klein, S. Adrenaline, cortisol, norepinephrine: the three major stress hormones, explained. *Huffingt. Post* (2013).
